# Potential Pathogenetic Role of the D313Y Mutation in the *GLA* Gene in Anderson Fabry Disease: Two Case Reports

**DOI:** 10.3390/ijms26094400

**Published:** 2025-05-06

**Authors:** Antonella La Russa, Antonio Siniscalchi, Ardito Bonaventura, Domenico Di Noia, Teresa Valsania, Giovanni Stallone, Luciano Tartaglia, Concetta Chiapparino, Giovanni Di Rienzo, Giuseppe Coppolino, Davide Bolignano, Teresa Faga, Ashour Michael, Alberto Montesanto, Raffaele Serra, Michele Andreucci

**Affiliations:** 1 Nephrology Unit, Department of Health Sciences, “Magna Graecia” University, I-88100 Catanzaro, Italy; gcoppolino@unicz.it (G.C.); ashourmicheal@yahoo.com (A.M.); 2 Department of Neurology and Stroke Unit, Annunziata Hospital, I-87100 Cosenza, Italy; anto.siniscalchi@libero.it; 3 Department of Neurology and Stroke Unit, Della Murgia Fabio Perinei Hospital, I-70022 Altamura, Italy; bonaventura.ardito@asl.bari.it (A.B.); domenico.dinoia@asl.bari.it (D.D.N.); concetta.chiapparino@asl.bari.it (C.C.); 4 Department of Nephrology and Dialysis, ASL “Guglielmo da Saliceto” Hospital Piacenza, I-29121 Piacenza, Italy; t.valsania@ausl.pc.it; 5 Nephrology Dialysis and Transplantation Unit, Advance Research Center on Kidney Aging (A.R.cK.A.), Department of Medical and Surgical Sciences, University of Foggia, I-71100 Foggia, Italy; stallonegio63@gmail.com (G.S.); tartaglialuciano@gmail.com (L.T.); 6 U.O.S.V.D. Patologia Clinica, Ospedale Della Murgia Fabio Perinei, I-70022 Altamura, Italy; giovanni.dirienzo@ausl.bari.it; 7 Department of Medical and Surgical Sciences, “Magna Graecia” University, I-88100 Catanzaro, Italy; dbolignano@unicz.it (D.B.); rserra@unicz.it (R.S.); 8 Renal Unit, Campus “S. Venuta”, “Renato Dulbecco” University Hospital, I-88100 Catanzaro, Italy; teresa_faga@yahoo.it; 9 Department of Biology, Ecology and Earth Sciences, University of Calabria, I-87036 Rende, Italy; alberto.montesanto@unical.it

**Keywords:** *GLA*, mutation, D313Y, VUS, Fabry disease, polymorphisms

## Abstract

Anderson Fabry disease (AFD) is an X-linked hereditary lysosomal abnormality that causes the accumulation of glycosphingolipids in body fluids and tissues, leading to progressive organ damage and a shortened life span. More than 1000 mutations in the *GLA* gene have been identified, promoting many different clinical pictures. For this reason, diagnosing AFD can be difficult, especially because of the great diversity of atypical clinical presentations that can simulate the disease. Some of these variants of the *GLA* gene have been described as non-pathogenic. For example, the D313Y variant is one of the most controversial, even if there are several case reports of D313Y patients presenting with signs and symptoms consistent with AFD without any other etiological explanation. This work aimed to clarify whether the presence of the D313Y variant affects α-Gal A activity and causes AFD symptoms and organ involvement in two patients from different families. The presence of the D313Y variant resulted in clinical manifestations of AFD in both patients and a decrease in alpha-galactosidase activity in the male patient. Two patients (one female and one male) from two unrelated families were examined. Sequencing of all seven *GLA* exons and the adjacent 5′ and 3′ exon–intron boundaries identified the D313Y variant in exon 6, as well as the genetic variation g.1170C>T in the flanking 5′ UTR in patient 1 only. Our results suggest that the D313Y variant is causative for the disease and that the clinical phenotype can be enhanced by the presence of other variants modulating protein expression.

## 1. Introduction

Anderson Fabry disease (AFD) is an X-linked hereditary lysosomal abnormality that causes the accumulation of glycosphingolipids in body fluids and tissues, leading to progressive organ damage and a shortened life span. AFD occurs in both males and females and can be classified into classical and late-onset phenotypes. In classical AFD, α-galactosidase A (α-Gal A) activity is absent or severely reduced, and the disease symptoms develop early and may affect multiple organs. Conversely, in late-onset AFD, α-Gal A activity remains present, and clinical manifestations are mainly confined to the heart. Individualized treatment goals are necessary because of the variable phenotypic and patient characteristics and the wide range of disease severity in AFD [1]. Heterozygous females show phenotypic variability depending on both the inactivation of the X chromosome and the type of variant. In these patients, the disease has a late onset, with organ involvement and enzymatic activity always being normal [2]. This heterogeneity in clinical presentation and enzymatic function has highlighted the need for more refined approaches to diagnosis and therapy. In this context, the meta-analysis conducted by Citro et al. [3] provides valuable insight by characterizing the structural and functional consequences of a wide range of missense mutations in the *GLA* gene. Their work supports a graduated diagnostic model and emphasizes the relevance of personalized therapeutic strategies, particularly in patients with non-classical or atypical variants. As a consequence of the phenotypic variability, the diagnosis of AFD is often difficult, and the underdiagnosis or misdiagnosis of AFD can delay the start of the specific treatment, thereby affecting the prognosis. Once a diagnosis is made, biochemical and molecular genetic analysis and subsequent cascade genotyping of family members can reveal greater numbers of affected individuals, often at younger ages than would otherwise be diagnosed [4]. Besides the more than well-known 1000 mutations, many variants still have an unknown significance with equivocal implications for AFD diagnosis and therapeutic management. A much-debated issue is the pathogenesis of the D313Y variant (exon 6, c.937 G/T). D313Y was defined as a pseudo-deficient allele, associated with reduced enzymatic activity and instability of the mutant enzyme at a neutral pH. The D313Y variant was first described in hemizygotes with the classic phenotype in association with other variants of the *GLA* gene, and it was suspected that they increased the expression of the disease [5]. D313Y has been considered a benign variant or a variant of unknown significance (VUS) recently, specifically due to the hypothesis that LysoGb3 levels are a biomarker of FD, which are usually not elevated in patients carrying this variant. However, several published papers describe patients with the D313Y variant exhibiting phenotypic manifestations attributable to FD, without any other clinical or laboratory explanation. Studies to date, however, have shown that other pathogenetic mechanisms could explain the clinical features of these patients [6]. In addition to VUS, many polymorphic variants of the *GLA* gene have been described, but their association with AFD is still unclear [7]. Specifically, some studies have shown that the g.1170C>T polymorphism (rs2071225) located in the 5′ untranslated region (UTR) is associated with reduced expression of *GLA* protein and neurological manifestations [8]. In this article, we present two families with different phenotypes harboring the D313Y variant.

## 2. Detailed Case Descriptions

### 2.1. Methodology

Two patients (1 female and 1 male) from two unrelated families were examined. The diagnosis of AFD was confirmed by the clinical features, brain MRI, cardiac MRI, and the results of the molecular and genetic analyses. Written informed consent was obtained before molecular analysis and publication. α-Galactosidase activity was determined from filter paper dried blood spots (Brunker Maxis Impact, Bremen, Germany). Lyso-Gb3 was assayed using tandem mass spectrometry on dried blood spots at Archimed Life Science GmbH (Vienna, Austria). For genetic analysis, the coding exons and flanking intronic regions of the GLA gene were amplified from purified genomic DNA. The products were purified with the Big Dye X Terminator Purification Kit (Thermo Fisher Scientific, Waltham, MA, USA) and resolved using the ABI 3500xL Genetic Analyzer. The data were analyzed using ABI Data Collection software (version 3.0, Thermo Fisher Scientific), Sequencing Analysis software (version 5.2, Thermo Fisher Scientific), and SeqScape software (version 2.6, Thermo Fisher Scientific). Sequences were compared with the reference DNA sequence (GenBank Accession: NM_000169.3).

### 2.2. Molecular Findings

**Figure 1:**
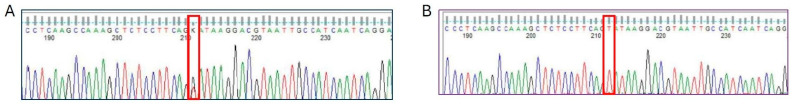
Sequencing analysis of c.937 G/T (D313Y) in the *GLA* gene (DNA): (**A**) heterozygote patient affected by the c.937 G/T; (**B**) hemizygote patient affected by the c.937 G/T.

Sequencing of all seven *GLA* exons and the adjacent 5′ and 3′ exon–intron boundaries identified the D313Y variant in exon 6 (Figure 1), as well as the genetic variation g.1170C>T in the flanking 5′ UTR in patient 1 only. The alpha-galactosidase activity in leukocytes was within the normal range in both patients (14.9 µmol/L/h), whereas in patient 2, it was reduced by 30% in the plasma (22 nmol/mg). Plasma lyso-Gb3 levels, an additional marker of AFD, were within normal limits (0.9 ng/mL). [7]

### 2.3. Characteristics of the Patients

#### 2.3.1. Patient 1

**Figure 2:**
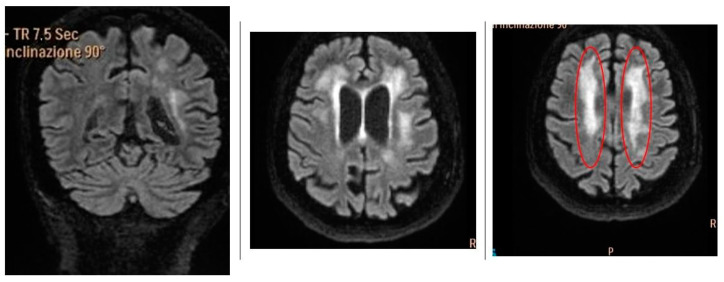
Axial fluid-attenuated inversion recovery images showing large chronic ischemic lesions and multiple white matter hyperintensities in bilateral cerebral hemispheres (red circles).

A 49-year-old woman had been complaining of burning pain and paresthesia in her hands and feet for several years. The patient’s son reported that the patient had progressive difficulty walking for approximately 4 to 5 months until she became completely bedridden. For this reason, in February 2020, the patient presented to the Neurology Stroke Unit Hosp. of the Murgia “F. Perinei” of Bari, Italy. At the time of admission, the physical examination showed mild dysarthria, hyposthenia of the lower limbs, and hyperreflexia of the osteotendinous reflexes of the lower limbs. The bilateral Babinski reflex test was positive. MRI of the brain was performed, and the patient’s clinical symptoms required the performance of appropriate diagnostic procedures, including biochemical and genetic analyses. Brain MRI showed multiple disseminated, hyperintense T2 signal changes in the bihemispheric periventricular subcortical white matter, posterior arm of the internal capsule, bilaterally, in the midbrain, and middle cerebellar peduncles (Figure 2). MRI of the spinal cord was normal.

Cerebrospinal fluid (CSF) analysis showed normal immunotyping as determined using flow cytometry and dementia biomarkers (amyloid beta (1–42), total tau, and hyperphosphorylated tau). However, no CSF-specific oligoclonal bands were detected. Manual cytological examination of cerebrospinal fluid revealed no malignant cells. The serum angiotensin-converting enzyme level was normal. Serum antibodies against aquaporin-4 water channel, thyroid peroxidase, thyroglobulin, glutamic acid decarboxylase, and GQ1b, as well as neuronal antibodies (anti-amphiphysin, anti-Ri, anti-Yo, anti-Hu, anti-CV2/CRMP5, anti-Ma2/Ta, anti-NMDA, LGI-1, and GAD) and anti-cardiolipin, were all negative. Screening for antinuclear and extractable antinuclear antibodies was negative. Screening for *Notch3* gene mutations consistent with cerebral autosomal dominant atherosclerosis with subcortical infarcts and leukoencephalopathy (CADASIL) was negative. Cerebral MR angiography and catheter angiography were normal, with no evidence of vasculitis. Electrocardiography revealed sinus bradyarrhythmia, and an echocardiogram showed concentric left ventricular hypertrophy with dynamic left ventricular outflow tract obstruction. Serum creatinine levels were within the normal range, and urine protein levels were slightly increased. Diagnostic investigation revealed no evidence of any (of the following) inflammatory, metabolic, degenerative, or congenital diseases in particular, and none of the classic risk factors for white matter lesions (WMLs) were present (Table 1).

Subsequent molecular genetic testing identified two different mutations in the *GLA* gene. The first was a missense variant c.937G>T (p.D313Y) in exon 6 of the gene, which has been associated with non-classical or atypical phenotypes of AFD. The second mutation, g.1170C>T, was located in the flanking 5′ UTR of the *GLA* gene. Although the pathogenicity of p.D313Y remains debated, the clinical context supported its potential disease relevance. A family pedigree was constructed, and Fabry-specific genetic testing was performed on available relatives, all of whom tested negative for the disease. In summary, the diagnosis of AFD in this patient was based on the combination of suggestive clinical signs (neuropathy, WMLs, and cardiac involvement) and the identification of *GLA* variants (p.D313Y and g.1170C>T) in the absence of alternative diagnoses.

#### 2.3.2. Patient 2

**Figure 3:**
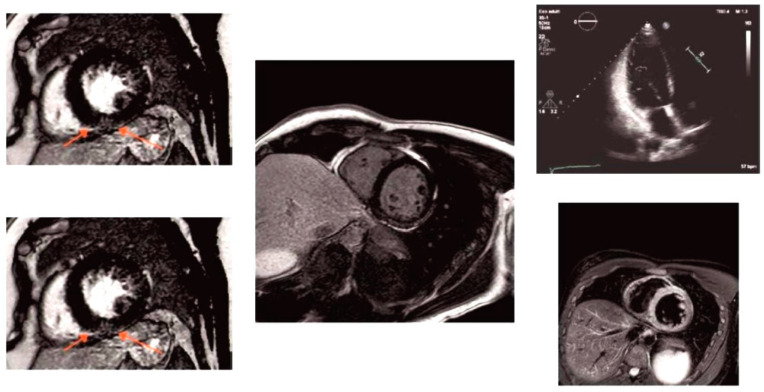
Cardiac magnetic resonance (MRI) showed late gadolinium enhancement of the inferior junctional and inferior wall in the middle segment (red arrows).

A 45-year-old man with a history of hypertension since childhood and chronic leg pain after road traffic polytrauma was a frequent user of Non-Steroidal Anti-Inflammatory Drugs (NSAIDs). He was first admitted to the neurology department (Guglielmo da Saliceto Hospital, Piacenza, Italy) in 2020 with a diagnosis of lacunar stroke in small vessel disease without embolic or atherosclerotic cause. In June 2021, the patient experienced acute coronary syndrome and was treated with percutaneous transluminal angioplasty and stenting of the anterior descending coronary artery. A consensual decline in renal function was noted (plasma creatinine: 2.18 mg/dL). The patient had another small lacunar ischemic stroke in October 2021. During his neurological recovery, the patient underwent an ophthalmological examination, which did not reveal the presence of cornea verticillate. In March 2023, the patient was admitted to the neurology department due to a recurrence of ischemic encephalopathy. A deterioration in renal function was also noted, with a creatinine level of 5.25 mg/dL. Transthoracic echocardiography revealed hypertrophic non-obstructive cardiomyopathy (SIVd 20 mm) with a normal biventricular ejection fraction. Cardiac magnetic resonance (MRI) showed late gadolinium enhancement of the inferior junctional and inferior wall (Figure 3).

Although renal biopsy was not feasible due to the elevated bleeding risk associated with dual antiplatelet therapy, and a skin biopsy proved inconclusive, the patient’s multi-organ involvement—recurrent strokes, progressive renal impairment, and hypertrophic cardiomyopathy—raised strong clinical suspicion for AFD. As a result, targeted biochemical testing was performed, revealing reduced α-galactosidase A enzyme activity in the plasma (22 nmol/mg) and a normal range in leukocytes (14.9 µmol/L/h). This biochemical finding, in the context of the patient’s clinical phenotype, was highly suggestive of AFD. Subsequent molecular genetic analysis confirmed the presence of the p.D313Y missense variant in the *GLA* gene, a variant historically classified as being of uncertain significance but increasingly associated with atypical or non-classical Fabry phenotypes. Following genetic counseling, all available family members underwent genetic testing for AFD after informed consent was obtained. All tested individuals were negative for Fabry-related variants. Given the early age of onset, progressive multi-organ dysfunction, and the presence of a *GLA* variant with potential clinical significance, enzyme replacement therapy (ERT) with beta agalsidase (105 mg every 15 days, 1 mg/kg) was initiated in August 2023. Since the initiation of ERT, the patient has remained free of new neurological or cardiac events, and renal function has stabilized, with serum creatinine improving to 3.3 mg/dL following dietary and supportive management. In summary, the diagnosis of AFD in this patient was based on a suggestive clinical phenotype (recurrent strokes, cardiac hypertrophy, and renal dysfunction), biochemical confirmation of low α-galactosidase A activity, and the identification of a potentially relevant *GLA* variant (p.D313Y), supporting the clinical decision to initiate specific therapy.

## 3. Discussion

To date, there is extensive literature describing patients with a Fabry phenotype carrying the D313Y variant. The D313Y variant is currently classified as a variant of unknown significance (VUS) with residual α-Gal activity. However, there is increasing evidence that it may be pathogenic. The prevalence and phenotypic characteristics of carriers of the *GLA* D313Y variant were highlighted in the study by Palaiodimou et al. [5]. Their results showed that the D313Y variant appears to correlate with an atypical, mild, late-onset phenotype of Fabry disease with a predominance of neurological involvement. However, other studies have shown that in some cases, the involvement of the kidneys and heart, as well as the neurological system, may be the cause of AFD. This suggests that this variant may also be the cause of other organ damage in carriers [9]. The confirmation of renal involvement comes from the Santostefano group [10], whose clinical and histological findings in four D313Y patients showed renal involvement, pyknotic mitochondria, endothelial activation, small vacuoles, especially in podocytes and tubules, microvacuoles containing multi-lipid droplets and lipofuscin, arteriosclerosis, and arteriolar hyalinosis. Also of interest is the multicenter study conducted in Greece, where 5 out of 14 patients with the D313Y mutation were identified with a definite diagnosis of FD. This has led to an estimated prevalence of the D313Y mutation in FD of more than 35%, whereas the frequency in the general population is estimated to be less than 1% [4]. Our cases are in line with recent studies that have reported an association of D313Y with other typical manifestations of AFD, such as peripheral neuropathy, hypertrophic cardiomyopathy, renal failure, or stroke, associated with a mild late-onset phenotype [11]. This study shows that in reality, female patients with AFD do not always have less severe symptoms [12]. Heterozygotes with one healthy inactivated allele tend to have a significantly higher clinical severity score than women with random inactivation of both X chromosomes. In women with X-linked disorders, the clinical manifestations may be biased in favor of the normal allele. This means that the manifestations of the disease in heterozygous women are usually mild and have a slower rate of progression. Studies have shown that methods of determining X-chromosome inactivation (XCI) asymmetry may not be sufficient to fully explain the manifestations of FD in women and that other events such as methylation processes should be considered. The methylation process was investigated in a study of 36 women in which disease severity was correlated with methylation sites. The study showed a clear correlation between the severity of the phenotype, the accumulation of lysoGb3, and the methylation of the normal allele, as detected by non-digestion with methylation-sensitive restriction enzymes [13]. Phenotypic differences can also be influenced by the external environment and other genetic variants that are modulators of protein expression. Currently, the detection of mutations in the *GLA* gene is considered essential for the clinical diagnosis of FD, often overlooking the fact that variants in intronic regions can alter the expression pattern of the *GLA* gene [4,5]. In fact, unlike mutations in coding regions that affect peptide sequences, those in intronic sequences are not predictable, even though efficient splicing of pre-mRNAs generally depends on conserved intronic sequences. In addition, splicing functionality can be further modified by splicing enhancers or suppressors and by sequences within exons and introns. Because of this complexity, it is not easy to understand the effect of a genomic variation on splicing without performing functional studies. For this reason, intronic variants of *GLA* often remain unidentified because they are not routinely assessed via gene sequencing, and the prevalence of AFD may, therefore, be underestimated [14]. The two genetic variants found in trans in this study—c.937 G/T (p. D313Y) and c.1170C>T—occurred simultaneously in patient 1. Functional studies have shown that the T allele of the c.1170C>T SNP causes a significant decrease in the transcriptional activity of *GLA* as well as in the binding of the protein to DNA [8]. Molecular studies have also shown that the c.1170C>T SNP allele causes altered binding of Transcription Factor EB (TFEB), which controls lysosomal biogenesis by positively regulating genes belonging to the Coordinated Lysosomal Expression and Regulator (CLEAR) network, which also regulates autophagy [15]. In addition, dysregulation of TFEB has been implicated in the development of many pathological conditions, and further evidence suggests that TFEB may be a point of convergence for multiple signaling pathways and, thus, modulate other important biological processes, such as cellular senescence, DNA repair, endoplasmic reticulum (ER) stress, and lipid metabolism, as well as processes related to WNT signaling [16]. Schelleckes et al. have shown that in patients with neurological manifestations typical of AFD without a *GLA* mutation, the SNP substitution c.1170C>T can cause the disease. These data suggest that *GLA* 5′UTR polymorphisms may be modulators of α-Gal A expression. Two trans variants in the *GLA* gene have rarely been reported, and the clinical significance of these two variants in AFD remains unclear. Although a functional impact on the protein’s 3D structure for D313Y mutation can be hypostasized, the location of the c.1170C>T SNP in the 5′ untranslated region (5′UTR) of the *GLA* gene suggests a possible impact on *GLA* expression and protein availability. For these reasons, we can speculate that patients carrying both mutations ultimately present a much more compromised clinical picture and protein behavior. Of course, further studies are needed to obtain more conclusive results. The clinical findings in patient 2 support the hypothesis that the D313Y mutation may be associated with later presentation and not only with neurological involvement. Based on our experience, it is imperative to continue measuring leucocyte enzymatic activity when plasma enzymatic activity remains normal. As an alternative, the detection of glycosphingolipid accumulation could be useful, but myocardial and renal biopsies are associated with a high risk of bleeding, especially in patients on anticoagulation or anti-aggregation therapy. In view of this, we suggest considering initiating therapy in patients with the D313Y mutation who show significant organ involvement. Further studies are needed to elucidate the biological and clinical implications of these observations, particularly to clarify the effect of these polymorphisms in individuals carrying *GLA* variants associated with high residual enzyme activity, with mild or no AFD clinical phenotypes.

In conclusion, this report presents two clinically relevant and genetically distinct Fabry disease cases that underscore the complexity of genotype–phenotype correlations. In the first case, we describe a unique female patient carrying two *GLA* variants in trans (p.D313Y and c.1170C>T), suggesting a possible synergistic effect between a non-coding regulatory variant and a missense VUS, potentially supporting a two-hit model of pathogenesis. In the second case, a male patient with a hemizygous p.D313Y mutation exhibited progressive multi-organ involvement, reinforcing emerging evidence that this variant may not be entirely benign and can justify enzyme replacement therapy in selected patients. Both cases highlight the clinical importance of integrating genetic data with phenotypic and imaging findings, particularly in non-classical or borderline presentations. Furthermore, they emphasize the limitations of relying solely on variant classification in therapeutic decision making. Collectively, these findings contribute novel insights into the clinical impact of under-recognized *GLA* variants and support the need for a more nuanced, individualized approach to diagnosis and treatment in Fabry disease.

## Figures and Tables

**Table 1:** Exclusion of risk factors and triggering factors for white matter lesions in Patient 1.

	Sample/Applications	Diagnostic/Risk Factors	Results
Neurological	Cerebrospinal fluid	✓ Beta-amyloid (1–42) ✓ Total tau ✓ Hyperphosphorylated tau ✓ Oligoclonal bands ✓ Anti-MOG and AQP-4 antibodies ✓ Anti-ganglioside and anti-onconeural antibodies	Normal
	Cerebral MR angiography Catheter angiography	Cerebral vasculitis	Normal
Cardiac	ECG		Sinus bradyarrhythmia
Renal	Serum	eGFP++, proteinuria/albuminuria	Normal

## Data Availability

Data are unavailable due to privacy restrictions.

## Abbreviations

The following abbreviations are used in this manuscript:

α-Gal A	α-galactosidase A
VUS	Variant of unknown significance
UTR	5′ untranslated region
MRI	Magnetic resonance imaging
CSF	Cerebrospinal fluid
WMLs	White matter lesions
NSAIDs	Non-Steroidal Anti-Inflammatory Drugs
XCI	X-chromosome inactivation
TFEB	Transcription Factor EB
CLEAR	Coordinated Lysosomal Expression and Regulator
CADASIL	Cerebral Autosomal Dominant Arteriopathy with Subcortical Infarcts and Leukoencephalopathy
